# Hemodynamic patterns in obesity associated hypertension

**DOI:** 10.1186/s40608-018-0190-8

**Published:** 2018-04-16

**Authors:** Catarina Santos, Pedro Marques da Silva

**Affiliations:** 1 Serviço de Nefrologia, Hospital Amato Lusitano, Unidade Local de Saúde de Castelo Branco, 6000-085 Castelo Branco, Portugal; 2 Faculdade de Ciências da Saúde, Universidade da Beira Interior, Covilhã, Portugal; 3 Núcleo de Investigação Arterial, Serviço de Medicina Interna, Centro Hospitalar de Lisboa Central, 1169-1024 Lisbon, Portugal

**Keywords:** Obesity, Hypertension, Impedance cardiography, Body mass index, Cardiac index, Systemic vascular resistance index

## Abstract

**Background:**

Obesity is a well-known condition of resistant hypertension (HT). Insights to the hemodynamic patterns that characterize obesity related hypertension may help guide therapeutic adjustments and shorten time to HT control.

**Methods:**

We performed a retrospective analysis of 202 patients followed at our Hypertension Clinic with the diagnosis of primary HT and who performed an impedance cardiography (ICG) test. Obtained data was analyzed to identify differences between obese and non-obese patients and to identify predictors of uncontrolled HT (≥ 140 and/or ≥ 90 mmHg) in obese patients.

**Results:**

One hundred patients were male (49.5%) and average age 54.6 ± 13.9 years. Average systolic and diastolic pressures were 136.5 ± 22.4 mmHg and 82.9 ± 5.1 mmHg, respectively. The average BMI was 28.9 ± 5.1 Kg/m^2^. Seventy one patients (35.1%) had systolic arterial pressure (AP) ≥140 mmHg and 45 patients (22.3%) diastolic AP ≥90 mmHg. BMI correlated with systolic and diastolic AP (Pearson’s coefficient 0.235; *p* < 0.001 and 0.163; p < 0.001, respectively). Obese patients presented increased cardiac index (CI) (*p* < 0.001), left cardiac work index (LCWI) (*p* < 0.001) and systemic vascular resistance index (SVRI) (*p* < 0.001) but reduced systemic arterial compliance index (SACI) (*p* < 0.001). Obese patients with uncontrolled HT had greater BMI (p < 0.001), CI (p < 0.001) and SVRI (p < 0.001) but lower SACI (p < 0.001) and LCWI (p < 0.001). In multivariate analysis, however, only CI remained predictive, conferring a risk 1.47 higher of uncontrolled HT.

**Conclusions:**

Obese patients, including those with uncontrolled HT, had increased CI and SVRI. The only predictor of uncontrolled HT, however, was CI, suggesting that the obese present a state of hyperinotropy and may benefit of “add-on” or increase treatment with beta-blockers.

## Background

The complex interplay between obesity and hypertension has been a matter of interest and debate for a long time. In the last decade several new insights into the pathophysiology linking obesity and hypertension have led to a better understanding of the mechanisms that beneath this association: insulin resistance, activation of the renin-angiotensin-aldosterone system (RAAS), dysfunctional adipose tissue and deregulated synthesis of adipokines, increased activity of the sympathetic nervous system, overproduction of inflammatory cytokines and obstructive sleep apnea are among some of these factors [1]. Together, these neuro-endocrine imbalances contribute to vascular and endothelial dysfunction, impaired pressure natriuresis and sodium excretion, increased cardiac output and changes in systemic vascular resistance and arterial compliance [2]. In this respect, the measurement of hemodynamic parameters may be a valuable tool for the management of obese hypertensive patients, not only to identify the main abnormalities associated with elevated blood pressure (increased CI, SVRI or fluid volume) [3] but also to help guide therapeutic adjustments according to the spectrum of hemodynamic changes identified [4].

It is also worthwhile to note that obesity is one of the most frequent causes of resistant hypertension, and identification of major hemodynamic abnormalities may help shorten the time to achieve blood pressure control and reduce global cardiovascular risk [3].

While invasive procedures are not adequate for ambulatory patients, non-invasive monitoring may be easily implemented using accurate and reproducible techniques like impedance cardiography [5]. Previous studies have shown the value of this technique in tailoring hypertension treatment in individual patients [6] and several trials have used ICG in large cohorts of hypertensive’s to identify factors associated with resistant hypertension [7] or to better achieve blood pressure control in patients with uncontrolled hypertension [3, 8]. We aimed to identify hemodynamic variables associated with poor blood pressure control in a population of obese hypertensive patients using ICG and to categorize the main changes in hemodynamic parameters associated with high BMI.

## Methods

### Study patients

We performed a retrospective analysis of 202 patients who attended our Hypertension Clinic and performed an ICG test for one of the two following clinical criteria: as part of the initial work-up at the Hypertension Clinic or follow-up of variations in hemodynamic variables after adjustments in anti-hypertensive therapy. All the patients were followed at our department, agreed to perform the exam and gave informed consent to participate. The exam was performed as outpatients and the ICG exams were not selected based on any time frame. Definition of obesity was based on World Health Organization criteria (BMI ≥ 30 Kg/m^2^) and respective grades: grade 1 with BMI between 30 and 34.9 Kg/m^2^, grade 2 with BMI between 35 and 39.9 Kg/m^2^ and grade 3 with BMI ≥ 40 Kg/m^2^. The study was approved by the Ethics Committee of Centro Hospitalar de Lisboa Central. All patients had been diagnosed with primary hypertension and no cases of secondary hypertension were included. All the patients selected had on the day of the exam AP > 100/60 mmHg under anti-hypertensive treatment; as we only based in one ICG exam per patient we excluded the one’s that at the time of the test were hypotensive, mostly because they might develop hemodynamic adaptation to lower levels of AP. Uncontrolled AP was defined as systolic AP ≥ 140 mmHg and/or diastolic AP ≥90 mmHg on the day of examination. Limitations for ICG were also observed and patients with severe aortic insufficiency, permanent pacemakers, atrial fibrillation and ventricular extrasystole were not included in this analysis. Even if the situations of high BMI may constitute a technical limitation to the ICG test, we did not exclude patients with grade 3 obesity. In the literature we can see that the ICG test is not extensively validated in patients whose weight is above 342 pounds [2] (~ 155 kg) due to limitations in signal detection. Nevertheless, in our cohort the highest weight registered was 127 kg, a value under the threshold considered.

On the day of the examination, weight and height data were collected and the BMI calculated. Blood pressure was obtained according to the current recommendations [9] using an oscillometric semiautomatic sphygmomanometer and reported as the average of 2 readings taken with 2 min intervals. The size of the bladder was adjusted to the arm circumference, using a larger bladder (arm circumference 31–40 cm) in obese patients. Situations of white coat hypertension could not be completely excluded because ambulatory blood pressure measurements were not available for all the patients studied.

### ICG and determination of hemodynamic variables

ICG, a non-invasive method of hemodynamic monitoring, is based on Ohm’s relationship [10]. It measures the instantaneous variations of an electrical sign across the thoracic cavity [2], which are then translated in variations in blood flow through the great vessels to yield the effective stroke volume [10]. Because of the differential properties to conduct current between blood and other biological tissues like muscle or bone, variations in electrical impedance essentially represent variations in stroke volume and net content of blood in the thoracic aorta [2]. Thus, when TFC rise, thoracic impedance falls since the results of impedance are expressed as the reciprocal of TFC [11].

All ICG exams were performed by trained technicians of our Hypertension Clinic and data was obtained during a scheduled visit. Patients rested for 5 to 10 min before the procedure and then blood pressure was measured with the patient in the supine position, with head elevated at 45 degrees. Then, paired electrodes were placed in each side of the neck and thoracic cavity [2] to detect the variations in the electrical sign. The data obtained (blood pressure values and ICG signals) were processed and stroke volume calculated.

The remaining variables produced by ICG output were subsequently analyzed according to a summarized classification previously published by others [12]:

1. Variables that express cardiac work: stroke volume, cardiac output, cardiac index and left cardiac work index
2. Variables that express fluid status: thoracic fluid content
3. Variables that express systemic vascular resistance: systemic vascular resistance and systemic arterial compliance index

Systemic arterial compliance (SAC) was defined as (10 x supine stroke volume)/ supine pulse pressure and systemic arterial compliance index (SACI) as (10 x supine cardiac index)/ supine pulse pressure. A SACI < 0.1 suggested arterial stiffness.

### Statistics

Considering sample size and distribution of patients through the different categories of weight classification, with under-representation of patients with grade 2 and 3 obesity, we performed weight adjustment using SPSS 17 (version 17, Chicago, Illinois). With this statistical approach we aimed to correct for the lack of representativity of specific patient groups, namely patients with grade 2 and 3 obesity. Considering also estimates from the World Health Organization (http://www.euro.who.int/en/health-topics/noncommunicable-diseases/obesity/data-and-statistics) for the prevalence of overweight and obesity for European Union countries of 30–70% and 10–30%, respectively, we saw that in our sample the proportion of overweight (38%) and obesity (37%) was very similar, not approximating the distribution of BMI in the population, reason why we performed weight adjustment of the variable BMI. Statistical analysis presents values for the different hemodynamic variables for “normal weight”, “overweight” and “obesity” and also for the three grades of obesity. T-test was used to compare “non-obese” (normal weight plus overweight) and “obese patients” and obese patients with “controlled” and “uncontrolled hypertension” (values of AP ≥140/90 mmHg on the day of the examination). Finally, using logistic regression analysis analysis and weight-adjusted hemodynamic variables (CI, LCWI, SVRI and SACI) we searched for predictors of uncontrolled hypertension in obese patients. The level of significance was defined to *p* < 0.05.

## Results

One hundred patients were male (49.5%) and average age 54.6 ± 13.9 years. Average systolic and diastolic pressures were 136.5 ± 22.4 mmHg and 82.9 ± 5.1 mmHg, respectively. The average BMI was 28.9 ± 5.1 Kg/m^2^: 46 patients (22.7%) had BMI between 20 and 24.9 Kg/m^2^, 78 (38.6%) between 25 and 29.9 Kg/m^2^ and in 75 patients (37.1%) BMI was ≥30 Kg/m^2^. According to gender the average values of BMI were 28.5 ± 4.5 Kg/m^2^ for men and 29.5 ± 5.4 Kg/m^2^ for women (*p* < 0.001). Seventy one patients (35.1%) had systolic AP ≥140 mmHg and 45 patients (22.3%) diastolic AP ≥90 mmHg. BMI correlated with diastolic AP (Pearson’s coefficient 0.163; *p* < 0.001) but the association was stronger for systolic AP (Pearson’s coefficient 0.235; *p* < 0.001). There was also a positive correlation between obesity and uncontrolled AP (Pearson’s coefficient 0.138; *p* < 0.001), although this correlation was weak.

No adverse effects secondary to the ICG procedure were reported.

Table 1 presents the values of hemodynamic variables according to weight categories “normal weight”, “overweight” and “obesity” for the cohort studied. Although no statistical differences were found in CI, LCWI, SVRI and SACI (*p* > 0.05) between normal weight and overweight patients we observed that differences became more pronounced with increasing BMI. In this way, when comparing non-obese (normal and overweight patients) with obese we observed an increase in CI (*p* < 0.001), LCWI (*p* < 0.001) and SVRI (p < 0.001) but reduction in SACI (*p* < 0.001) (Table 2).

**Table 1:** Values of hemodynamic variables according to BMI categories normal weight, overweight and obesity

	Normal weight (*N* = 46)	Overweight (*N* = 78)	Obesity (*N* = 75)
BMI (Kg/m^2^)	23.4 ± 1.2	27.3 ± 1.4	34.2 ± 3.6
SAP (mmHg)	133.3 ± 26.6	132.6 ± 19.1	142.9 ± 21.5
DAP (mmHg)	82 ± 13.8	81.6 ± 9.2	85 ± 10.7
CO (L/min)	5.2 ± 1.1	6.5 ± 6.3	6.7 ± 6
CI (L/min/m^2^)	3.0 ± 0.6	3.0 ± 0.5	3.4 ± 0.6
LCW (Kg.m)	6.6 ± 1.9	6.9 ± 1.6	8.1 ± 2.1
LCWI (Kg.m/m^2^)	3.9 ± 1.2	3.8 ± 0.8	4.5 ± 4
SV (mL)	80.2 ± 19.3	87 ± 19.6	89.3 ± 23
TFC (/KOhm)	30.3 ± 3.7	30.2 ± 3.7	29.9 ± 3.8
SVR (dyne.sec.cm^− 5^)	1495 ± 439	1425 ± 490	1361 ± 390
SVRI (dyne.sec.cm^− 5^.m^2^)	2514 ± 609	2521 ± 588	2643 ± 706
SAC	17.2 ± 6.1	18.6 ± 6.7	16.5 ± 6.2
SACI	0.65 ± 0.2	0.65 ± 0.2	0.62 ± 0.5

Values of hemodynamic variables presented as average ± standard deviation. (CI: cardiac index; CO: cardiac output; DAP: diastolic blood pressure; LCW: left cardiac work; LCWI: left cardiac work index; SAP: systolic arterial pressure; SV: stroke volume; SVR: systemic vascular resistance; SVRI: systemic vascular resistance index; TFC: thoracic fluid content; SAC: systemic arterial compliance; SACI: systemic arterial compliance index)

**Table 2:** Values of hemodynamic variables from non-obese and obese patients

	Non-obese patients (*N* = 124)	Obese patients (*N* = 75)	*p*
BMI (Kg/m^2^)	25.8 ± 2.3	34.2 ± 3.6	< 0.001
SAP (mmHg)	132.9 ± 21.8	142.9 ± 21.5	< 0.001
DAP (mmHg)	81.8 ± 10.9	85 ± 10.7	< 0.001
CO (L/min)	6 ± 5.1	6.7 ± 6	< 0.001
CI (L/min/m^2^)	3.0 ± 0.5	3.5 ± 3.4	< 0.001
LCW (Kg.m)	6.8 ± 1.7	8.1 ± 2.1	< 0.001
LCWI (Kg.m/m^2^)	3.8 ± 0.9	4.5 ± 4.1	< 0.001
SV (mL)	84.4 ± 19.7	89.3 ± 22.9	< 0.001
TFC (/KOhm)	30.2 ± 3.7	29.5 ± 3.8	0.021
SVR (dyne.sec.cm^− 5^)	1449 ± 471	1361 ± 389	< 0.001
SVRI (dyne.sec.cm^− 5^.m^2^)	2517 ± 590	2643 ± 706	< 0.001
SAC	18.1 ± 6.5	16.5 ± 6.2	< 0.001
SACI	0.65 ± 0.2	0.62 ± 0.5	0.003

Values of the hemodynamic variables presented as average ± standard deviation. *p* obtained from T-Test for independent samples. Level of significance defined to *p* < 0.05

However, we also noted that these variations were neither progressive throughout the 3 grades of obesity nor statistically significant and, even if we observed an increase in stroke volume and cardiac output with increasing BMI, these changes reflect mainly a greater body surface area and inherently a physiological larger cardiac output. Besides, the small number of patients with grade 3 obesity limits the interpretation of these variations throughout the three grades of obesity (Table 3).

**Table 3:** Values of hemodynamic variables from obese patients according to the degree of obesity

Grades of obesity	Grade 1 (*N* = 49)	Grade 2 (*N* = 21)	Grade 3 (*N* = 5)
SAP (mmHg)	143.5 ± 21	149 ± 19.5	145 ± 19.6
DAP (mmHg)	85.6 ± 12	86.9 ± 8.2	85.3 ± 6.6
CI (L/min/m^2^)	3.7 ± 4.6	3.0 ± 0.5	3.4 ± 0.6
LCWI (Kg.m/m^2^)	4.9 ± 5.5	4.1 ± 1	4.6 ± 0.8
TFC (/KOhm)	29.4 ± 3.4	29.2 ± 4.5	30.6 ± 4.7
SVRI (dyne.sec.cm^− 5^.m^2^)	2685 ± 680	2840 ± 830	2298 ± 234
SACI	0.66 ± 0.6	0.52 ± 0.2	0.62 ± 0.2

To further characterize AP in obese patients and hemodynamic factors associated with poor blood pressure control we searched for differences between patients with controlled and uncontrolled HT (≥140/90 cut-off) using only weight-adjusted variables. After univariate analysis we found that uncontrolled obese patients had greater BMI (*p* < 0.001), CI (*p* < 0.001) and SVRI (*p* < 0.001) (Table 4) but lower SACI (*p* < 0.001) and LCWI (*p* < 0.001). In multivariate analysis (Table 5), however, only CI remained predictive, conferring a risk 1.47 higher of uncontrolled hypertension. The other weight adjusted variables (LCWI, SVRI and SACI) were not associated with increased risk of uncontrolled HT. Specifically, SVRI, although with statistical significance, had an odds < 1, suggesting that it doesn’t contribute to uncontrolled HT in this specific cohort.

**Table 4:** Values of hemodynamic variables from obese patients with controlled and uncontrolled hypertension

	AP < 140/90 mmHg (*N* = 36)	AP ≥ 140/90 mmHg (*N* = 39)	*p*
BMI (Kg/m^2^)	34.1 ± 3.5	35.1 ± 3.9	< 0.001
SAP (mmHg)	127 ± 9.4	157 ± 19.4	< 0.001
DAP (mmHg)	79.1 ± 6.3	90.2 ± 11.1	< 0.001
CO (L/min)	6.2 ± 1.5	7.1 ± 8.1	< 0.001
CI (L/min/m^2^)	3.1 ± 0.5	3.7 ± 4.7	< 0.001
LCW (Kg.m)	7.6 ± 2.1	8.5 ± 2	< 0.001
LCWI (Kg.m/m^2^)	4.7 ± 5.8	4.3 ± 1	< 0.001
SV (mL)	93.7 ± 25	84.3 ± 20.1	< 0.001
TFC (/KOhm)	29.7 ± 3.4	29.4 ± 4.2	0.005
SVR (dyne.sec.cm^− 5^)	1200.8 ± 290	1505 ± 410	< 0.001
SVRI (dyne.sec.cm^− 5^.m^2^)	2339 ± 467	2915 ± 770	< 0.001
SACI	0.67 ± 0.2	0.59 ± 0.6	< 0.001

Values of the hemodynamic variables presented as average ± standard deviation. *p* obtained from T-Test for independent samples. Level of significance defined to *p* < 0.05

**Table 5:** Logistic regression analysis to identify hemodynamic variables predictive of uncontrolled HT in obese patients

	Unstandardized Coefficients B	Odds ratio (β)	*P*	95% Cl for B
Constant	0.112		0.005	0.034; 0.190
CI (L/min/m^2^)	0.210	1.470	< 0.001	0.196; 0.223
LCWI (Kg.m/m^2^)	0.000	−0.004	0.806	−0.004; 0.003
SVRI (dyne.sec.cm^− 5^.m^2^)	0.000	0.315	< 0.001	0.000; 0.000
SACI	−1.452	−1.383	< 0.001	− 1.554; − 1.351

R^2^ 0.392; ANOVA *p* < 0.001

Regarding SACI, the odds associated with uncontrolled HT was − 1.383, pointing to reduced compliance as a predictor of difficult to control AP. Besides, SACI was reduced not only in obese patients with uncontrolled HT but also with increasing weight.

## Discussion

Obese hypertensives present increased values of CI and SVRI in comparison with non-obese patients. However, the most important hemodynamic determinant of uncontrolled systolic and/or diastolic AP is increased CI. Taking into account that AP is the product of CO and SVR, and using weight-adjusted variables, our results show that in the obese hypertensive patient variations in CI are more important in determining the level of AP.

In obese patients SAP and DAP were greater than in non-obese, reflecting that in these patient population there is increased risk of uncontrolled HT for which seem to contribute both CI and SVRI. There was also increased LCWI in the obese patients. However, this variable, which results from the product of mean arterial pressure and CI, reflects more the higher oxygen demands of the heart in these circumstances than increased cardiac performance itself. Probably, this is also the reason why, in multivariate analysis, LCWI was not predictive of uncontrolled HT as it relates more with coronary ischemic threshold and does not represent a true measure of contractility.

In this way, changes in CI seem to be the most appropriate to guide therapeutic adjustments in the hypertensive obese population studied. These results are in accordance with others previously published [13–16], where obesity is frequently associated with increased cardiac output and CI to accomplish with the higher metabolic demands. In this context, the choice of anti-hypertensive medication should focus more on drugs like beta-blockers or central acting agents [3, 7] that may decrease cardiac output and the effort performed by the myocardium to pump blood through the vessels. Concerns about weight gain related to beta-blockers should be considered, since several trials have documented an association between beta-blockers, weight gain and accumulation of visceral adiposity [17, 18]. Central agonists, to a lesser extent, may be a second choice mainly due to a more unfavorable profile of side effects.

The SVRI values, with a normal range between 1970 and 2390 dyne.sec.cm^− 5^.m^2^, were systematically high across all groups of patients (normal weight, overweight and obesity). The differences were significant between non-obese and obese and became even more pronounced when the cut-off ≥140/90 mmHg was established. In this regard, decreasing SVRI, which is an indirect measure of cardiac afterload, should be part of the anti-hypertensive regimen using agents like blockers of the renin-angiotensin-aldosterone system (RAAS) and calcium channel blockers.

Remarkably, however, SVRI was not predictive of uncontrolled HT in the obese patient in multivariate analysis. Although it is expected that vasoconstriction contributes to high levels of blood pressure, several reasons may explain why we didn’t observe that in our cohort: the increase in AP levels was not paralleled by the increase in SVRI and the absence of knowledge on current anti-hypertensive medication, specially with drugs that interfere with arterial tonus, does not to allow to interpret the heterogeneity of SVRI values found. However, it is also recognized that a blunted reduction of peripheral resistance, despite the increased stroke volume and CI, is a common finding in obesity [14, 16] that may contribute to the high levels of SVRI observed. Some factors that may explain this blunted reduction in the tonus of resistance vessels include activation of the sympathetic nervous system and release of substances from the adipocytes [1] leading to high levels of arterial resistance.

Considering SACI, we observed lower levels in the obese hypertensive, especially in the patients with uncontrolled AP and, in multivariate analysis, this variable was predictive of uncontrolled AP. Lower levels of SACI, representing an indirect measure arterial stiffness, were associated with greater risk of blood pressure ≥ 140/90 mmHg. Considering that SACI results from the ratio of CI and pulse pressure it is reasonable to expect, especially in those with higher levels of blood pressure, higher values of pulse pressure and, concomitantly, lower levels of SACI.

It is also worthwhile to note that obese patients, in general, and those with uncontrolled blood pressure had lower values of TFC. Although these data do not point to hypervolemia as a determinant factor to high blood pressure in this cohort, the absence of data concerning use of diuretics limits the interpretation of these values. In the model of multivariate analysis we didn’t included this variable, as expressed in Kohm and not adjusted to body surface area, doesn’t reflect volume/m^2^. Nevertheless, variations of TFC may be very sensitive and consistent with volume expansion [19]. The usefulness of TFC measurements have been previously documented in other studies, proving beneficial in hypertensive patients to help guide diuretic adjustments in those who present with increased chest fluid volume and hypervolemia [19–21] and also in established heart failure, to identify patients at risk for acute decompensation [22, 23].

There are limitations to this work since its retrospective nature and several relevant data missing. One of the most important in this context concerns information on current anti-hypertensive medication on the date of the ICG examination, which we were not able to fully collect due to technical problems concerning data archives and lack of data on waist circumference and abdominal obesity. BMI, although reflecting weight excess and being the basis for obesity grading, does not allow to identify patients with predominantly central adiposity. The evidence in the literature regarding the pathogenic role of abdominal fat is vast and the contribution of insulin resistance, oxidative stress and deregulated synthesis of hormones by adipose tissue for cardiovascular disease is well documented [24–28]. In our cohort we observed high prevalence of overweight and obesity and, probably, stratifying patients according to values of waist circumference would allow improvement in identifying the hemodynamic pattern associated with this type of obesity, more frequently associated with HT.

In obese patients, trends in CI and SVRI are clearly distinct from the one’s we found in lean subjects (which presented a predominance of increased SVRI in spite of CI) and it would be important to investigate if central obesity is associated with extreme values of the considered hemodynamic variables or, in alternative, with variations in other parameters that would impose changes in anti-hypertensive therapy. It should also be mentioned that when considering abdominal obesity, in addition to waist circumference, other elements should also be taken into consideration, namely the differential composition of abdominal fat (subcutaneous versus visceral fat) and the proportion of fat-free mass. While some studies support the importance of fat-free mass as a major determinant of the variations in SV and CO to accomplish with greater metabolic demands in the obese patient [14, 29] others suggest the predominant role of fat mass in the disturbance of hemodynamics [13]. Given the intense metabolic activity of adipocytes it is expected that the several molecules they secrete may interfere with systemic hemodynamic, inducing a state of high CI and blunted decrease in SVRI. Future research including a greater number of obese patients, complemented with data of body composition in terms of relative fat distribution, would allow for a better understanding of hemodynamic patterns associated with different types of obesity.

It is also important to refer two other limitations: first, patients with cardiac arrhythmias were excluded from this study and, in the general hypertensive population they represent a large proportion of patients. In this regard, as the study of hemodynamic in this specific subset is unreliable with ICG, non-invasive determination of CI and SVRI is compromised in these patients. Second, when classifying patients with controlled versus uncontrolled HT we based exclusively on the values of AP obtained in the day of examination. Information about number of medications in each group, time of follow-up in the Hypertension Clinic or time to achieve blood pressure control would better describe the differences between the two groups.

## Conclusions

Tailored anti-hypertensive therapy for the obese patient should focus in the balance between CI and SVRI in each patient. In our cohort, anti-hypertensive drugs aimed to modulate cardiac index, mainly beta-blockers, and decrease SVRI like inhibitors of the RAAS and calcium channel blockers seem the most appropriate to control blood pressure. Increased levels of CI contribute to higher risk of uncontrolled HT in the obese, situation in which adjustment of drugs that reduce hyperinotropy may improve and shorten time to blood pressure control.

## Abbreviations

AP: Arterial pressure
BMI: Body mass index
CI: Cardiac index
CO: Cardiac output
DAP: Diastolic blood pressure
HT: Hypertension
ICG: Impedance cardiography
LCW: Left cardiac work
LCWI: Left cardiac work index
RAAS: Renin angiotensin aldosterone system
SAP: Systolic arterial pressure
SVR: Systemic vascular resistance
SVRI: Systemic vascular resistance index
TFC: Thoracic fluid content
